# Implementation of a referral pathway for cancer survivors to access allied health services in the community

**DOI:** 10.1186/s12913-023-09425-4

**Published:** 2023-05-04

**Authors:** Lahiru Russell, Rebecca McIntosh, Carina Martin, Wee Kheng Soo, Anna Ugalde

**Affiliations:** 1 School of Nursing and Midwifery, Deakin University, 1 Gheringhap Street, Geelong, Australia; 2 Centre for Quality and Patient Safety Research in the Institute for Health Transformation, Deakin University, 1 Gheringhap St, Geelong, VIC 3220 Australia; 3 Centre for Quality and Patient Safety Research – Eastern Health Partnership, 2/5 Arnold St, Box Hill, Victoria 3128 Australia; 4 healthAbility, Box Hill, Victoria Australia; 5 Eastern Health, Box Hill, Victoria Australia; 6 Eastern Health Clinical School, Monash University, Box Hill, Victoria Australia

**Keywords:** Survivorship, Community-based health, Allied health, Service utilisation, Referral pathway

## Abstract

**Background:**

The growing demands for multidisciplinary cancer survivorship care require new approaches to address the needs of people living after a cancer diagnosis. Good Life–Cancer Survivorship is a self-management support survivorship program delivered by community allied health (AH) services for people diagnosed with cancer. A pilot study established the benefits of Good Life–Cancer Survivorship to help survivors manage their health and wellbeing in the community health setting. This study expanded the program to four community health services and evaluated the implementation outcomes of the referral pathway to the survivorship program.

**Methods:**

Eligible cancer survivors attending hospital oncology services were referred to the survivorship program. Data was collected between 19/02/2021-22/02/2022 and included allied health service utilisation, consumer surveys, and interviews to understand consumer experience with the referral pathway. Interviews and focus groups with hospital and community health professionals explored factors influencing the referral uptake. Implementation outcomes included Adoption, Acceptability, Appropriateness, Feasibility, and Sustainability.

**Results:**

Of 35 eligible survivors (mean age 65.5 years, SD = 11.0; 56% women), 31 (89%) accepted the referral. Most survivors had two (n = 14/31; 45%) or more (n = 11/31; 35%) allied health needs. Of 162 AH appointments (median appointment per survivor = 4; range = 1–15; IQR:5), 142/162 (88%) were scheduled within the study period and 126/142 (89%) were attended. Consumers’ interviews (n = 5) discussed the referral pathway; continuation of survivorship care in community health settings; opportunities for improvement of the survivorship program. Interviews with community health professionals (n = 5) highlighted the impact of the survivorship program; cancer survivorship care in community health; sustainability of the survivorship program. Interviews (n = 3) and focus groups (n = 7) with hospital health professionals emphasised the importance of a trusted referral process; a holistic and complementary model of care; a person-driven process; the need for promoting the survivorship program. All evaluations favourably upheld the five implementation outcomes.

**Conclusions:**

The referral pathway provided access to a survivorship program that supported survivors in self-management strategies through tailored community allied health services. The referral pathway was well adopted and demonstrated acceptability, appropriateness, and feasibility. This innovative care model supports cancer survivorship care delivery in community health settings, with clinicians recommending sustaining the referral pathway.

**Supplementary Information:**

The online version contains supplementary material available at 10.1186/s12913-023-09425-4.

## Introduction

Cancer survival rates have increased substantially worldwide due to early detection and advances in treatments [1]. Cancer survivorship care encompasses the prevention or treatment of recurrences and additional cancers, the management of additional chronic conditions including chronic effects of treatments such as psychosocial and economic burden, and the promotion of healthy lifestyle behaviours and adherence to long-term treatment and follow-up care [2]. The diversity of care needs in specific survivor groups calls for care coordination between medical specialties, allied health professionals, and expert nurses [3]. However, current survivorship care is mainly managed in acute specialist-led clinics [4] with limited care coordination across allied healthcare services. With increasing pressures on limited acute care resources and growing demands for multidisciplinary survivorship care, new approaches are urgently needed to address the needs of people living after a cancer diagnosis [5]. The Clinical Oncology Society of Australia highlights the importance of coordinated, integrated access to allied health professionals and self-management support in models of cancer survivorship care [6].

Our team conducted a pilot study to establish and evaluate a referral pathway to a community-based cancer survivorship program called the Good Life-Cancer Survivorship program [7]. The study reported on 25 referrals made over five months where 18 patients were followed through the referral process. Interviews with health professionals involved in the referral pathway highlighted the benefits of integrated care coordination involving community-based nursing and allied health professionals, and demonstrated the value of a community health setting to support cancer survivors in managing their health and well-being [7]. Needs around training information and communication were also emphasised by hospital and community health professionals. These findings informed this larger implementation project to expand the delivery of the survivorship program across four community health settings. Central to this expansion was the capacity building of the community allied health workforce to support cancer survivors in self-management and implementing the referral pathway.

The aim of this study was to evaluate the implementation of the referral pathway to the four community health settings. Specifically, this paper reports on the adoption, acceptability, appropriateness, and feasibility of the referral pathway to a community survivorship program.

## Methods

This study used a mixed methods design. A prospective cohort study was used to evaluate the referral process and community-based allied health services utilisation, and nested semi-structured interviews explored the challenges and enablers of the survivorship program uptake.

### The Good Life Cancer Survivorship (GL-CS) program

GL-CS is a community-based model of survivorship care delivered by allied healthcare services and offers a person-centred and coordinated model of care promoting lifestyle and behaviour change [7]. An initial assessment with a community-based Integrated Care Coordinator confirms the survivor’s medical conditions and areas of concern, followed by discussions about survivorship care planning and service options to address care needs. Eligible participants in the survivorship program had completed curative-intent cancer treatment, had metastatic hormone sensitive cancer, were receiving maintenance immunotherapy, or were unable to participate in intensive ambulatory oncology rehabilitation. Those with bone metastases or severe osteoporosis with stable lesions who would benefit from an exercise-based program were also eligible. People were excluded if they had acute care needs or were medically unstable.

### Participant recruitment and data collection

All referrals to the survivorship program were made from hospital-based oncology services to four community health services, namely healthAbility (previously Carrington Health), Inspiro, Access Health & Community, and EACH all located in the eastern suburbs of Melbourne (Australia). The same method of participant recruitment described in the pilot study [7] was employed in this study. Briefly, eligible patients were identified by hospital-based oncology health professionals and referred to one of the participating community health services. All referrals were then screened and triaged by the project manager at healthAbility to confirm eligibility and transfer the referral to the appropriate community health service. Eligible patients were contacted and seen by an integrated care coordinator at their community health centre.

The four types of data collection used are described below:

1. *Health service utilisation data*: A data collection tool was developed in Microsoft Excel to track survivors referred to community health services. Characteristics of survivors (age, gender, socioeconomic status, cancer, cancer treatment, and time from last cancer treatment) and the type and number of community health services utilised were recorded. Postcodes were used to derive a proxy for participants’ socioeconomic status using the Socio-Economic Indexes for Areas [8], and level of accessibility to service centres using the Accessibility/Remoteness Index of Australia [9]. Both indices were obtained from the Australian Bureau of Statistics. Data was entered by the site-specific coordinator using information received by hospital staff who referred their patients. Referrals were made by via fax or email using the program specific referral form (available as an electronic or paper-based form). Following the initial call, the site-specific coordinator booked all subsequent allied health appointments.
2. *Consumers’ survey*: A survey explored participants’ experience with their community health service. Questions related to participants’ perception of healthcare provider (HCP) knowledge about patient’s health history (e.g. “They knew about your medical history including your cancer diagnosis and treatment”); participant-HCP communication (e.g. “They explained things in a way that was easy to understand”); person-centred care (e.g., “You had an opportunity to talk about ongoing problems related to your cancer”); shared healthcare planning (e.g. “They helped you make a plan to improve your health”); administrative process (e.g. ” It was easy to book an appointment”). Possible answers were given on a 5-point Likert scale ranging from 1- strongly disagree to 5- strongly agree. Open-ended questions explored aspects of care that participants found helpful or needed improvement, and whether they had already or were intending to change their health behaviour following their appointments at their local community health service.
3. *Consumer interview*: Consumers’ who completed the survey were invited to a semi-structured interview to further explore their experience with the survivorship program. Questions aimed to elicit consumers’ perspectives on how the health services work and communicate information to them and how they felt about it.
4. *Health professionals’ interviews and focus groups*: Community health professionals were interviewed using a semi-structured interview guide to explore their perspectives about the referral pathway and delivery of the survivorship program. Questions aimed to elicit views on the feasibility of the survivorship program, its suitability for the target group, and whether further training or support was required. Confidence in delivering the program was assessed using a structured questionnaire where questions were read out to participants who responded against a 5-point Likert scale (1 = Not at all confident to 5 = Highly confident). Scores between 1 and 3 indicated low confidence.

Focus groups were scheduled with hospital health professionals to explore their experience referring people to the survivorship program and how the process could be improved. Questions aimed to elicit knowledge about the survivorship program, experience with the referral process, and suggestions for improvement and sustainability.

All interviews and focus groups were recorded, transcribed, and thematically analysed. This involved verbatim reading of transcribed quotes and assigning codes which were then structured according to overarching themes [10].

**Fig. 1:**
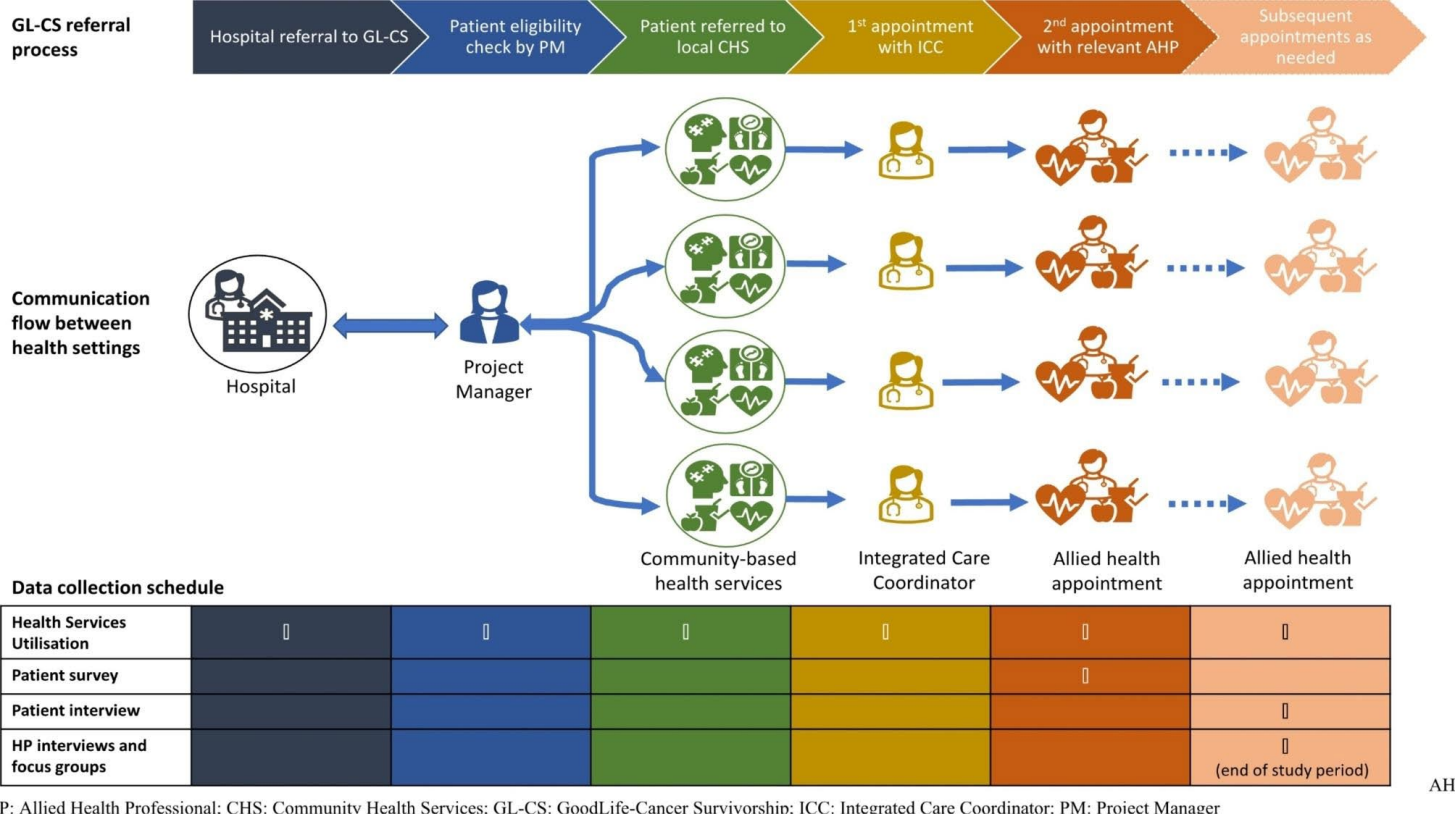
GL-CS referral pathway, communication flow between health settings, and data collection schedule

Fig. 1 summarises the referral pathway to the survivorship program, the communication flow between health settings and the data collection schedule.

### Implementation of the survivorship program

Strategies used to implement the survivorship program were coded against principles of management (Planning, Organising, Leading, Monitoring) [11] and external Influences as outlined in a previous systematic review [12]. Table 1 provides a detailed summary of the domain-specific strategies used to implement the referral pathway to the survivorship program.

**Table 1:** Implementation strategies used for GoodLife-Cancer Survivorship

**Planning** - Strategies in relation to establishing goals and creating courses of action to achieve them
• *Consultation with and engagement of* hospital and community health professionals and consumer representative
• *Support from senior leadership* of the participating community health services as the project was sponsored by the general manager of one of the community health services
• *Support from frontline staff* in hospital and community health settings
• *Review of administration support and training needs* of all participating community health services
• Referral to the survivorship program was previously evaluated in a *pilot study*
• *Multidisciplinary implementation team* involving hospital and community health professionals and managers, academic researchers, and a consumer representative
• Guidance from a *steering group*, including hospital and community health service managers/executive, a consumer representative, cancer networks/agencies, and the Department of Health
• Adoption of Proctor’s *implementation framework*
• *Implementation was tailored* to each setting
• *Targeting and engaging* three additional community health services to deliver GL-CS
**Organizing** - Modifying structures, allocating resources including staff, delegating tasks to achieve goals established during planning
• *Upskilling and certification of key staff –* community health professionals were trained in cancer survivorship care, which involved prerequisite modules on cancer treatment protocols and information for use at the point of care, workshop format with content and discussion, and case studies.
• *Additional staff appointments*: o 0.5 EFT for a combined Senior Clinician/Project Manager role o in kind support from one of the community health services – Allied Health Assistant to coordinate local client system procedures, Marketing/Communication/Corporate Services o each community health service partner offset 0.2EFT salary for the Allied Health Assistants/Administrative Assistants
• *Delegation of new tasks* to existing staff
• *Appointment of program champions* in hospital specialist nurses
• *Designating a communication path* between hospital staff, the project manager, and community health staf*f*
**Leading** - Encouraging and enabling people to take effective action
• *Staff development -* Allied Health Assistants/Administrative Assistants were trained in project administration tasks
• *Development of promotional material* for recruitment purposes
• *Distribution of information* in forums/oncology grand rounds and site visits (as COVID permitted)
• *Adaptation of paper-based forms to electronic systems -* The referral form was available on the hospital intranet site, but not embedded in the hospital medical record (future development)
• *Communication with staff* about the implementation through site visits
• *Redeveloping* assessment, treatment, and referral practices
• Nurse champions and allied health staff acting as *change agents*
• *Financial incentives –* upskilling of community allied health professionals was fully covered
• *Negotiations with management in community health services* regarding the established chronic disease management model of care
**Monitoring** - Evaluating the execution of the plan and making adjustments to ensure goals are achieved
• *Audits* of data collection were completed monthly at each community health service
• *Supervision* of data collection and maintenance by the project manager
• *Feedback* on the program and its operations were collected through interviews and focus groups
**External influences** - Organisations and individuals external to the organisation exerting an influence on the intervention
• *Academics* involvement in the planning and evaluation process
• Government funding supported the project
• *Cancer-focused agencies* were consulted and actively involved at the steering committee and project team level
• *External non-academic integrated cancer service organisation* was involved in training staff
• *Clinicians and oncologists* influenced the selection of target populations

### Outcome measures

Implementation outcomes were selected from Proctor’s implementation outcomes framework [13], namely acceptability, adoption, appropriateness, and feasibility (Table 2). These outcomes were measured using a mixed method approach.

**Table 2:** Implementation outcomes

Implementation domains	Operational definitions for the purposes of this study
Acceptability	Perception among stakeholders that the GL-CS referral pathway is satisfactory.
Adoption	Uptake of the referral pathway by referring health professionals and participants.
Appropriateness	Relevance^^^ of the referral pathway and GL-CS to stakeholders.
Feasibility	Extent to which referrals to GL-CS are issued by hospital staff and managed in community health settings.

^^^The referral pathway and GL-CS program are relevant when they meet patients’ healthcare needs and address a gap in service provision

## Results

### Participants’ characteristics

Between 19/02/2021 and 24/12/2021, 39 people were referred through the GL-CS referral pathway to participating community health services. Cancer care nurses referred most of the people (n = 28; 72%), followed by oncologists/ haematologists (n = 9; 23%), and allied health professionals (n = 2; 5%).

**Fig. 2:**
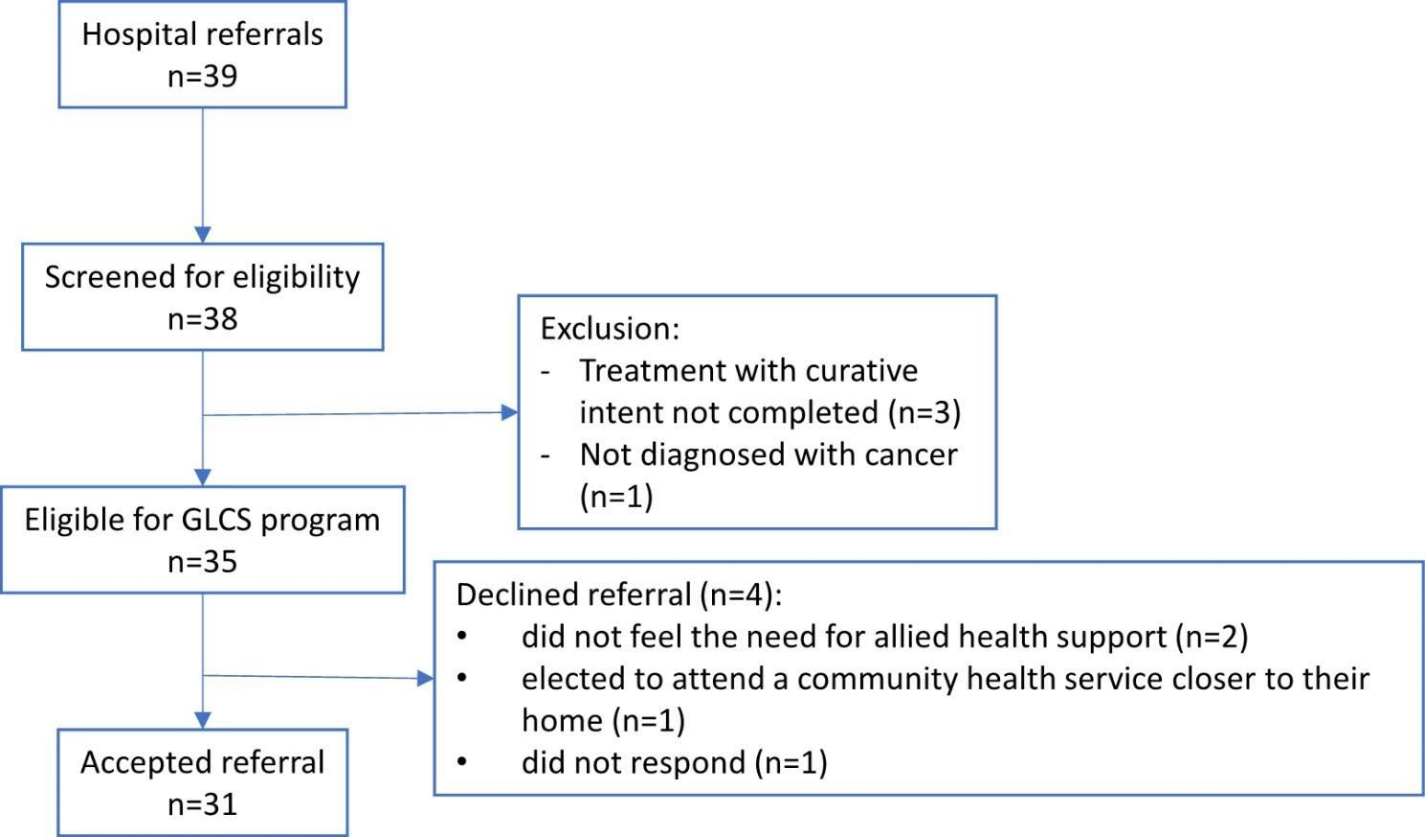
Participants’ referral diagram

People referred had a mean age of 65.6 years (SD 11.0), 22 (56%) were women, and 34 (87%) spoke English at home. One person was excluded due to an ineligible diagnosis (see Fig. 2) and was re-directed to general physiotherapy of the community health service. The mean years since cancer diagnosis of the 38 patients was 2.14 years (SD 3.26). Of the 38, 14 people (37%) were diagnosed with stage 4 cancer, and 27/38 (71%) were receiving cancer treatment at the time of referral. Table 3 provides additional information about the participants’ sociodemographic and clinical characteristics.

**Table 3:** Sociodemographic and clinical characteristics of people referred to GL-CS

**Sociodemographic characteristics (n = 39)**		
**Place of birth**	n	%
Oceania (Australia, NZ)	24	61
Europe (England, Italy, Netherlands)	7	18
Asia (Iran, Malaysia, Sri Lanka, China, Hong Kong)	5	13
Africa (Egypt, South Africa)	2	5
Unknown	1	3
**Language spoken at home**		
English	34	87
Other (Dutch, mandarin, Cantonese, Persian)	5	13
**Socio-economic Index for area** ^#^		
Quintile 1	0	0
Quintile 2	1	3
Quintile 3	3	8
Quintile 4	17	43
Quintile 5	18	46
**Accessibility/Remoteness index for Australia**		
Major Cities	37	95
Inner Regional	2	5
**Clinical characteristics (n = 38)**		
**Cancer type**		
Lung	11	29
Breast	11	29
Bladder	7	18
Other (Prostate, Colorectal,	9	24
**Cancer stage**		
Non-metastatic (Stage 0-III)	18	47
Metastatic (stage IV)	14	37
Unknown	6	16
**Time since diagnosis (years)**		
<= 1	17	45
> 1–3	12	31
> 3–5	6	16
> 5–10	3	8
**Type of treatment received**		
Immunotherapy	22	58
Surgery	19	50
Chemotherapy	18	47
Radiation therapy	11	29
Oral therapy	10	26

^#^Quintile 1 is the relatively most disadvantaged area, and quintile 5 is the relatively most advantaged area

A final cohort of 31 survivors were included in the study. Figure 2 describes participants’ referral process in further detail.

### Community health services utilisation

Between 19/2/21 and 22/02/2022, a total of 162 community allied health appointments were scheduled with a median of four appointments per cancer survivor (range:1–15; IQR:5). Of the 162 appointments made, 142 (88%) were scheduled by 21/01/2022, and 126/142 (89%) were attended. The remaining 20/162 (12%) appointments were scheduled beyond the study period and were not included in the analysis. Nearly half of those appointments were Integrated Care Coordination (61/126, 48%), followed by physiotherapy (24/126, 19%), dietetics (14/126, 11%), exercise physiology (12/126, 10%), counselling/psychology (9/126, 7%), podiatry (5/126, 4%), and occupational therapy (1/126, 1%).

The number of allied health needs identified per participant ranged from one service need for 6 participants (19%), two services need for 14 participants (45%), three services need for six participants (19%), and four services needed by five participants (16%). Due to health deterioration three (10%) participants were transferred back to acute care utilising existing clinical care practices to support the escalation of care. At the conclusion of the data collection period, six participants (20%) remained on waiting lists for allied health services.

Relevance to implementation outcome: Consistent GL-CS referrals by hospital health professionals and 89% people taking up the referral demonstrated successful **adoption** of GL-CS. The **appropriateness** of GL-CS was demonstrated as most people had two or more allied health needs.

### Consumers’ survey

Nine people (9/31; 29%) completed the online survey. Of this, one person (1/9; 10%) needed the help of a family member. Eight people (8/31; 26%) answered questions about their experience with their local community health service. All participants (8/8) strongly agreed that they had been treated with respect, most (7/8) ‘agreed’ to ‘strongly agreed’ to statements related to their health professional’s knowledge of medical and health history, their clear communication and respectful behaviour, their person-centred care and shared care planning approach, and the ease of the booking process (Supplemental material 1 - Consumer experience with local community health service). The overall experience of community health service use was ‘good’ to ‘great’ (6/8), ‘fair’ (1/8), and ‘unsure’ (1/8). Six participants (6/9; 67%) answered the open-ended questions about health behaviour changes and indicated they have or were considering making behaviour changes relating to: adopting new health care perspectives (“Taking on alternative ideas”), increasing physical activity (“…waiting for next appointment to do exercises. I would love to increase stamina and reduce tiredness”), increasing engagement and adherence to healthcare (“Can access caring, multiple services readily”; “By following the plan provided”; “Will be following up with GP for referrals for additional testing and alternative medications”), increasing own health knowledge (“…become more knowledgeable”).

### Consumer interviews

Five participants (5/9; 56%) agreed to be interviewed, among whom one used the help of an interpreter. Three themes were identified illustrating the use of and access to community health allied healthcare services for cancer survivorship:

1) *the cancer survivorship referral pathway* was perceived as a gateway to cancer survivorship allied healthcare services (“it seems that once I had the referral done as a cancer survivor person, I was able to get physio and podiatry and all of those things that I had tried to access probably 18 months ago.” pt4), a convenient continuation of care (“going to a place that’s closer to you but also easy, accessible” pt2), a person-driven process (“I self-referred” pt4) and a process that needs to be better communicated to people (“[they said] nothing, just someone from [CHS name] will call you” pt1).

2) *continuation of survivorship care in community health settings* was discussed in terms of coordinated and integrated care (“the community health have been able to offer me other things like, they put me in touch with the dental hospital " pt3), participant’s physical health and wellbeing improvement (“I would go to the bench at the back and I sit down and up without pushing up.“ pt2), affordable services (“I asked about community health services because I knew that I couldn’t afford private physio.“ pt4), and person-centred care (“they listened to me” pt2).

3) *opportunities for improvement of the survivorship program* included the provision of additional services (“you don’t offer general practice” pt3), optimising the length of sessions (“I think he ran out of time. I think they allow an hour. By the time you go through your medical history and the time you do everything else, and then you start doing your baseline exercises, it was too short. " pt1), and language barriers ([Interpreted] Because they [local CHS] have Chinese speaking staff. So, when they [local CHS] contact him, they contact him, and they are Chinese. So, if he’s tried to contact them [local CHS], like he might not get connected with the Chinese speaking staff so they can’t communicate…. when you call the centre, the one picks up the phone, normally they speak English and then he doesn’t know whether they speak Chinese and then it would be really hard if that person is like English only.” pt5). Additional quotes are provided in Supplemental material 2 – Consumer thematic analysis.

When asked about the impact of COVID-19 restrictions, participants did not express any concerns about attending their community health services as they had telehealth as an alternative approach and felt comfortable with the density levels at their centre; “going to the community health where there’s fewer people, that sort of stuff, not a problem with it”.

Relevance to implementation outcomes: The overall consumer satisfaction with GL-CS reported in the survey confirms **acceptability**. Referral to the program demonstrates **feasibility** as consumers valued the flexibility in accessing GL-CS.

### Health professional interviews

Five community allied health professionals were interviewed including three occupational therapists, one dietician, and one community health nurse. They had worked in community health settings for a median of four years (range: 9 months − 11 years; IQR: 3 years) and were all involved in the Integrated Care Coordination role. All respondents (n = 5) were confident in selecting and performing appropriate assessment, accessing reliable and high-quality information and resources for the consumer, and knowing what general services or supports were available for the consumer. However, some were less confident in providing evidence-based management or treatment (n = 3/5; 60%), understanding the cancer survivorship model of care (n = 2/5; 40%), knowing what cancer-specific services or supports are available for people (n = 2/5; 40%), and finding information about best practice clinical intervention (n = 2/5; 40%).

Three main themes emerged from the interviews:

*1) The impact of the GL-CS referral pathway* on the communication between acute and community health settings (“I think it’s really improved communication between the different health settings.” AHP3), empowerment of the cancer survivor across the two care settings(“I think having the care coordinator to prompt them, and give them those opportunities can sort of be a little bit of a light bulb moment for them” - AHP5), addressing a gap in healthcare (“[a process that] identifies people in hospital setting, that initial identification and having a place to refer those patients to meet those needs, I think is a great strength of the program” - AHP4), and access to coordinated and integrated care (“to have a really strong multidisciplinary approach. Being able to have a key point of contact for the client is a real strength as well.“ - AHP3).

2) *Cancer survivorship care in community health*. Cancer is perceived as a growing comorbidity in community health settings (“Yes, most clients have a cancer history, it’s quite common as a comorbidity” - AHP4), where access to allied health care is affordable and convenient (“The vast majority of our clients come from a low income, so if a client has financial hardship their fees can be waived.“ - AHP2) but dependent on consumer engagement and personal situation (“the personal characteristics of the client, I guess, rather than the complexity of their medical history or diagnoses or treatment” - AHP3).

3) *Sustainability of GL-CS* was discussed in the context of multi-level communication needs (“we haven’t had as much communication from our managers around the program … so that’s fallen on the care coordinators to educate the rest of the team about the program.“ - AHP3), ongoing training requirements to support additional integrated care coordination roles in community health services (“I would need ongoing professional development. “ - AHP1), considerations to streamline different funding schemes (“Each CHS has a slightly different funding source. So for us it has been about [figuring] out how to work things out for this program ” - AHP4), and workload management to address staffing issues (“the only real issue that we faced was just, yeah, trying to fit those clients into our regular diaries with our regular clients as well.“ - AHP5), work allocations and support. The possibility of expanding the program beyond cancer survivorship was also discussed (“there would be lots of clients in the Northern or Western regions that would definitely need that sort of support as well. So, it would be good to have community health centres around the whole of Victoria supporting this program” - AHP5).

More quotes are provided in Supplemental material 3.

One focus group and three semi-structured interviews were conducted due to scheduling difficulties with hospital health professionals. One inpatient social worker and six oncology nurses participated in either the focus group or interviews. Areas of expertise of the oncology nurses included prostate cancer, breast cancer, lung cancer, urology, and outpatient clinics. The median years of experience in acute care settings was six years (range: 6 months to 30 years; IQR = 7.75 years).

Four main themes emerged from the focus group and interviews:

1) *A holistic and complementary model of care.* GL-CS was described as a continuation of cancer survivorship care that is holistic, flexible, convenient, supportive, and complementary (“I’ve had a positive experience, and I think it’s really good to have something there that we can refer on to when we don’t have those services ourselves. " EH6). Additional service needs were also discussed regarding smoking cessation, social worker support, and difficulties in timely access to some services (“The only thing that I wish that they offered that they don’t is social work” EH 7).

2) *The referral process* was perceived as easy to apply. Sharing patient information called for caution and was guided by community health professionals (“I only provide that information if the physio or [project manager] has actually asked for it. " EH1). Participants acknowledged good communication flow between the two healthcare settings (“I’ve always had good communication from [community health services] after I’ve sent a referral " EH6) and reflected on ways to streamline the process within existing systems and implement a central point of contact beyond the study period (“All the referrals go to the one person, the intake person, who then streamlines them and sends them out to the appropriate people. Whether you had something like that might work.“ EH5). Participants discussed the possibility of broadening eligibility criteria (“We do have a lot of patients who are quite stable, but who will be on long-term treatments. So, I guess those sorts of patients would be great to be able to refer as well” EH6), the impact of COVID-19 on service disruptions and re-prioritisation (“this COVID time in acute health, everything seems to be about cutting back and not providing excess service” EH2), and the use of telehealth (“We don’t pick up a lot of the cues when things are done over the phone. Face-to-face, you do pick up a lot more” EH3).

3) *A consumer-driven process.* Participants acknowledged the central role of cancer survivors in the referral process (“it’s really whether they accept it or not… so as long as they’re receptive to [the referral], as long as they’re willing to partake in [the program]” EH5) and discussed how sex differences (“it’s interesting that sometimes the women are more likely to engage in wanting to be referred, but I find men are not wanting to be referred as often” EH3), the role of carers, the stage of recovery (“Until they’re at crisis point, they [patients with complex disease burden] often won’t accept the service or the help” EH7), and providing purpose for the referral (“it’s trying to encourage them that this is actually a really good service for them” EH5) influenced people’s decision to take up the referral.

4) *Promoting GL-CS* by raising awareness in acute care settings (“Making us aware of it is a good idea.“ EH3), in the community, among survivors, and by survivors was considered important (“I had a patient last week telling me of what a wonderful program it was, and she told me all the services and I must say, it woke me up again. " EH2). Maintaining awareness of GL-CS in acute settings through various pragmatic communication strategies (“I think putting the program out there regularly and always being in people’s faces or the doctors’ faces reminding them of the service. I think that is the best way to ensure that the program’s sustainable. " EH7), via consumer feedback and by increasing knowledge around cancer survivorship and services offered by GL-CS was regarded as an important promotion aspect for sustainability (“[I ask my patients] can you just keep us informed how you’re finding it, whether you’re…enjoying it, the benefits? Because that’s sort of information that I also need to be able to say to our other patients, look, we’ve had this person, he actually found it really beneficial for these reasons. This might actually work for you as well. " EH5).

Supplemental material 4 describes these themes in more detail.

Relevance for implementation outcomes: Health professionals demonstrated that referral to the program was **acceptable** as they reported perceived benefits of the GL-CS referral pathway, **appropriate** as cancer survivorship was recognised as a chronic condition in community health settings, and hospital and community health staff highlighted the importance of continued survivorship care in community health services, and **feasible** as GL-CS was successfully carried out within existing service systems, hospital staff perceived the referral process easy to action, and the Integrated Care Coordinator role was recognised to facilitate the process.

## Discussion

This study evaluated the implementation outcomes of a community-based multi-disciplinary cancer survivorship model of care that was expanded to four community health services following a previously reported initial pilot study [7]. The evaluation integrated data from the referral process in hospital settings to community health service utilisation, and consumers’ and health professionals’ experiences. Findings demonstrated the successful implementation of the referral pathway to the survivorship program with evidence for adoption, acceptability, appropriateness, and feasibility. The program and referral process were acceptable to consumers, health professionals, and referrers. The integration of the survivorship program into existing community health and chronic disease management programs demonstrated the feasibility and appropriateness of the program implementation across multiple community health services. Our study findings are in line with the principles outlined in the Australian Principle of Cancer Survivorship and support Principle 3 by providing evidence for a care pathway that is safe, and tailored for the individual circumstances and needs [14].

The Integrated Care Coordinator role was the most utilised service and pivotal to the coordination of allied healthcare services, and integration of care between health settings. The provision of effective care coordination within proven chronic disease management programs to help survivors’ transition from acute to community-based care is well supported [15]. Our study demonstrated the feasibility of implementing an integrated and coordinated chronic disease management role for cancer survivors in community health settings. The importance of this coordinator role was acknowledged by health professionals across hospital and community health settings. Hospital staff suggested maintaining awareness of the survivorship program in acute settings and community allied health staff recommended ongoing training in care coordination to support better care integration in community health settings. These recommendations are consistent with expert advice originating from a study that interviewed 27 implementation leaders and staff involved in the implementation of innovations in cancer survivorship [16]. Of the 16 factors perceived to influence sustainability, staff and organisational ‘buy-in’ for the program were outlined as necessary implementation prerequisites [16]. The central role of health professionals in referring to and implementing a survivorship program was further highlighted in an online survivorship care program that reported low uptake [17]; a major factor for the low uptake of the program was potential referrer’s lack of familiarity with the program and forgetting to refer people to the program [17]. Therefore, addressing the recommendations listed under theme 4 - “*Promoting GL-CS”* from hospital staff in the current study will likely support the continuation of the survivorship program and help maintain the referral pathway.

Most cancer survivors had two or more allied health needs identified. A multidisciplinary team is needed to address cancer and non-cancer conditions promote healthy lifestyle behavioural change [18]. The variety of healthcare expertise available in community health settings combined with specialist oncology care in hospital settings offers optimum value and quality care to consumers. It is noteworthy that multi-morbidity is common among survivors [19], and with improvement in cancer treatments, cancer is becoming more of a chronic disease. This is observed in our study where cancer is commonly perceived as a chronic comorbid condition in community health settings. People living after a cancer diagnosis are a growing consumer group for community health services focused on chronic disease management, and referral numbers are likely to increase [20, 21]. In addition, consumers in our study reported a preference for accessing care in the community health setting, which supports the expansion of community-based models of care to address current and future demands in cancer survivorship. Advocacy for a community-based funding model for cancer survivorship care is needed to utilise the chronic disease management expertise within the community health setting and build on existing workforce capabilities to optimise intrinsic capacity and functional ability and promote healthy ageing for cancer survivors [22].

It is important to note that our study period did not allow for a follow-up period and therefore we could not properly assess the sustainability of the survivorship program. However, as staff in community health settings have received training in survivorship care through this study, they now have the skills and knowledge to better understand and address the needs of people affected by cancer. Consumers in this study were all referred to the survivorship program by hospital staff and may have already had survivorship-related conversations with their specialist cancer nurse. Expanding awareness of and access to the referral pathway to general practitioners may be particularly relevant where survivors, on completing acute treatment, are not yet ready to consider and take up the next phase of survivorship care. The critical ongoing relationship with their general practitioner provides the opportunity for trusted referral pathways when the person is ready to take up this option.

## Conclusion

The implementation of a referral pathway to a survivorship program in community health settings has demonstrated an innovative care model for successful multidisciplinary cancer survivorship. Leveraging existing chronic disease management expertise within community health settings provided the basis for a coordinated and integrated approach to care. This study confirmed the feasibility and appropriateness of our survivorship model of care for replication across community health settings. It has provided access to relevant and needed allied healthcare services to cancer survivors, empowering them to actively manage their health and facilitate a transition from acute treatment to the maintenance of health. Community-based models of survivorship care offers a valuable approach to supporting people with cancer and reduce the burden on hospital-based oncology services, making them a valuable addition to cancer services.

## Electronic supplementary material

Below is the link to the electronic supplementary material.

Supplementary Material 1

Supplementary Material 2

Supplementary Material 3

Supplementary Material 4

## Data Availability

All data generated or analysed during this study are included in this published article and its supplementary information files.
