# Supplementary material for: Implementation of a referral pathway for cancer survivors to access allied health services in the community
Source: BMC Health Serv Res. 2023 May 4;23:440. doi: 10.1186/s12913-023-09425-4 (PMC10159668; doi:10.1186/s12913-023-09425-4)
Supplement: Supplementary file 1 — Supplementary Material 1 [file 12913_2023_9425_MOESM1_ESM.docx]

|  | Strongly agree | Agree | Not sure | Disagree | Strongly disagree |
| --- | --- | --- | --- | --- | --- |
|  | n (%) | n (%) | n (%) | n (%) | n (%) |
| 1. They knew about your medical history including your cancer diagnosis and treatment | 2 (25) | 5 (63) | 1 (13) | 0 (0) | 0 (0) |
| 1. You had an opportunity to talk about ongoing problems related to your cancer | 2 (25) | 5 (63) | 0 (0) | 1 (13) | 0 (0) |
| 1. You were able to talk about your needs after cancer | 3 (38) | 4 (50) | 0 (0) | 1 (13) | 0 (0) |
| 1. They spoke in a way you could understand | 4 (50) | 3 (38) | 0 (0) | 1 (13) | 0 (0) |
| 1. They explained things in a way that was easy to understand | 2 (25) | 5 (63) | 0 (0) | 1 (13) | 0 (0) |
| 1. They were sensitive to your needs and preferences | 3 (38) | 4 (50) | 0 (0) | 1 (13) | 0 (0) |
| 1. They treated you with dignity and respect | 5 (63) | 3 (38) | 0 (0) | 0 (0) | 0 (0) |
| 1. They gave you clear instructions about what you need to do after your visit | 3 (38) | 3 (38) | 2 (25) | 0 (0) | 0 (0) |
| 1. They helped you make a plan to improve your health | 2 (25) | 3 (38) | 3 (38) | 0 (0) | 0 (0) |
| 1. You felt confident that the plan and treatment provided will help you improve your health after cancer | 3 (38) | 3 (38) | 1 (13) | 1 (13) | 0 (0) |
| 1. It was easy to book an appointment | 2 (25) | 5 (63) | 1 (13) | 0 (0) | 0 (0) |
| 1. You would recommend your local Community Health Service to other cancer survivors | 5 (63) | 2 (25) | 1 (13) | 0 (0) | 0 (0) |

**Supplemental material 1 - Consumer experience with local community health service**
