# Supplementary material for: Implementation of a referral pathway for cancer survivors to access allied health services in the community
Source: BMC Health Serv Res. 2023 May 4;23:440. doi: 10.1186/s12913-023-09425-4 (PMC10159668; doi:10.1186/s12913-023-09425-4)
Supplement: Supplementary file 2 — Supplementary Material 2 [file 12913_2023_9425_MOESM2_ESM.docx]

**Supplemental material 2 – Consumer thematic analysis**

| **Themes** | **Sub-themes** | **Participant quotes** |
| --- | --- | --- |
| 1. Cancer survivorship referral pathway | 1. Greater access to cancer survivorship care | "I was going in for daily or monthly treatment, and then I got referred by the coordinator. She came out and asked did I still have back pain, et cetera, and I said yes, and she referred me." pt1 |
|  |  | "it seems that once I had the referral done as a cancer survivor person, I was able to get physio and podiatry and all of those things that I had tried to access probably 18 months ago." pt4 |
|  | 1. Convenient continuation of care | "going to a place that's closer to you but also easy, accessible" pt2 |
|  |  | " I hope it stays at CHS because they’re close" pt3 |
|  |  | "I can’t drive that far, so they will arrange either home visits or visits at my closer centre" pt4 |
|  | 1. Person-driven process | "I self-referred" pt4) |
|  |  | "I'm keen to get the next step because I want to improve. I want to improve my stamina" pt1 |
|  |  | "anything that could just well, strengthen me" pt2 |
|  |  | "[the nurse] suggesting I go through a questionnaire and pursuing that and following it up." pt3 |
|  |  | "I received treatment in the hospital, right, and then after that I was like resting at home and then I know that I will need some exercise or recovery treatment, like - to like get my life back to normal. That's why I asked the hospital and then they referred me to the community health service" pt5 |
|  | 1. Unclear purpose of referral | "If someone had said look – someone like you, in fact, had said there is a developing or developed protocol wherein you’ve had most of your cancer treatment. We’re consciously set up here to try and help you in other ways and look after you too in your post-cancer life. No one really said that. I just went along with it all. Clearly, there was good intent." pt3 |
|  |  | "nothing, just someone from [CHS name] will call you" pt1 |
|  |  | "Someone had given me a sheet of paper at [acute care] saying, we are helping you affect a bit of a transition in care and an expansion of care and exploring these areas and you go to[CHS] to do that. " pt3 |
| 1. Continuation of survivorship care in CHS | 1. Coordinated and integrated care | "the community health have been able to offer me other things like, they put me in touch with the dental hospital " pt3 |
|  |  | "He’s going to refer me for dry needling, so I had dry needling through a private physio and that helped a lot." pt4 |
|  |  | "he’s consulting with colleagues before my next appointment to see what the best plan is and to then liaise with the exercise physiologist so that any exercise program that’s designed isn’t going to exacerbate any problems that I have with the back and the leg." pt4 |
|  | 1. physical health and wellbeing improvement | "I would go to the bench at the back and I sit down and up without pushing up." pt2 |
|  |  | "I'm hoping it will improve and I won't fall asleep on the couch all the time and be tired all the time… [you attend community health services] to improve your livelihood, to improve your wellbeing," pt1 |
|  | 1. affordable service | "good dental treatment at a much lower cost" pt3 |
|  |  | "I asked about community health services because I knew that I couldn’t afford private physio." pt4 |
|  | 1. person-centred care | "He explained what he was doing. During my exercises for the baseline we had to stop because my oxygen in my blood had dropped down to 70. So, he stopped then he explained why he stopped. He explained everything and what he was doing all the way through and why he was doing it, which was good." pt1 |
|  |  | "they listened to me" pt2 |
| 1. Opportunity for GL-CS program improvement | 1. Additional services | "you don’t offer general practice" pt3 |
|  |  | "if they could bring in things like remedial massage, pampering, it’s nice to have a facial or have your nails done and just relaxation type of thing" pt4 |
|  | 1. Optimising the length of sessions | "Maybe get the patient to fill out the document and treatment and what they're looking for out of the physio, so is it just to cope with the pain, is it to help build stamina and pain management." pt1 |
|  |  | "I’ve got physio at the moment and poor guy, half-hour appointment and I was there for over an hour trying to work out the issue" pt4 |
|  |  | "I think he ran out of time. I think they allow an hour. By the time you go through your medical history and the time you do everything else, and then you start doing your baseline exercises, it was too short. " pt1 |
|  | 1. Language barrier | [Interpreted]” He's still waiting for them [local CHS] to contact him because they said if it's available [in person physiotherapy] then they would contact him but yeah I guess it's - he was still waiting.” pt5 |
|  |  | [Interpreted] Because they [local CHS] have Chinese speaking staff. So, when they [local CHS] contact him, they contact him, and they are Chinese. So, if he's tried to contact them [local CHS], like he might not get connected with the Chinese speaking staff so they can't communicate…. when you call the centre, the one picks up the phone, normally they speak English and then he doesn't know whether they speak Chinese and then it would be really hard if that person is like English only.” pt5 |
