# Supplementary material for: Implementation of a referral pathway for cancer survivors to access allied health services in the community
Source: BMC Health Serv Res. 2023 May 4;23:440. doi: 10.1186/s12913-023-09425-4 (PMC10159668; doi:10.1186/s12913-023-09425-4)
Supplement: Supplementary file 3 — Supplementary Material 3 [file 12913_2023_9425_MOESM3_ESM.docx]

**Supplemental material 3 – Thematic summary of community-based Allied Health Professionals interviews**

| Theme | Sub-theme | Code | AHP quotes |
| --- | --- | --- | --- |
| 1. The impact of GL-CS program | 1. Communication between acute and community settings |  | "I think it's really improved communication between the different health settings. In Eastern Health where the referrals are coming from and community health, there's a bit more of a link now and providing referrer feedback as well." - AHP3 |
|  |  |  | "It has sort of started to create good relationships with the hospital, and the staff at the hospital so that, moving forward, we’ve got a little bit more rapport with them, and getting more referrals in, which is always a good thing." - AHP5 |
|  | 1. Empowerment of cancer survivors | in acute healthcare setting | "for the client it’s really just about giving them that pathway to link in with health professionals that they perhaps previously never knew about or never thought about engaging with. So, it gives them that opportunity which I think is really great as well" - AHP5 |
|  |  |  | "I think someone in that cancer survivorship phase who doesn't have a good understanding or health literacy of the health care system would benefit more from this program. Those people who are still experiencing the side-effects of the cancer like isolation or deconditioning or fatigue. And just aren't sure where to go for support." - AHP4 |
|  |  | … but needs clear purpose | "he was unsure what the referral meant… he didn't think he needed it, he was feeling well" - AHP1 |
|  |  | in community health setting | "The good thing with GLCS is that you're revisiting overall health concerns, understanding what services to use and putting people in control of where they want to go. Moving from a health medical model to a support care type model. Hospital based care are very much "you need this, you need that" to go to treatment. In this model we're saying "what concerns do you have, what services do you need", so it's just putting people in control of their healthcare a bit more I think." - AHP1 |
|  |  |  | "I think having the care coordinator to prompt them, and give them those opportunities can sort of be a little bit of a light bulb moment for them" - HP5 |
|  |  |  | "people who have completed treatment and are sort of back in the community, or back at home, are definitely benefitting more from this sort of program only because they’ve got a little bit more time and energy to be able to focus on, you know, engaging with Allied Health Services" - AHP5 |
|  | 1. Addressing a gap in cancer survivorship care |  | "There is a gap in cancer survivorship care that this program fills … when their treatment finishes, they're discharged and that's kind of it. The support is gone, and they are left to figure things out for themselves ...their whole cancer experience has a physical and mental toll on them, so there is a need for a better pathway for a more coordinated care. They need support to adjust, manage and rehabilitate" - AHP2 |
|  |  |  | "[a process that] identifies people in hospital setting, that initial identification and having a place to refer those patients to meet those needs, I think is a great strength of the program" - AHP4 |
|  |  |  | "it would have even perhaps been up to the person themselves, if they identified their own issue, they would perhaps have to reach out to, you know, get their doctor to refer them to, I think the [unclear] or a psychologist or something like that, and I think a lot of clients – a lot of people don’t necessarily often initiate it themselves, they think they can just deal with it on their own…" - AHP5 |
|  | 1. Access to coordinated and integrated care |  | "So, you know, they perhaps previously wouldn’t have ever thought of seeing some of those health professionals, but because they’ve come in through this program, they now have the opportunity to link in with those people." - AHP5 |
|  |  |  | "Given the growing number of cancer survivors in the community, it’s important to address the needs that they have in order to prevent further deterioration of heath and to optimise their QoL and get back on track. So yeah very important to integrate in healthcare." - AHP2 |
|  |  |  | "having the time to speak to that client about all of their different needs and being able to sort of link them in with people that could help them." - AHP5 |
|  |  |  | "to have a really strong multidisciplinary approach. Being able to have a key point of contact for the client is a real strength as well." - AHP3 |
| 1. Cancer survivorship care in community health | 1. Cancer is a comorbidity |  | "In my current role I've had had clients with a cancer diagnosis, but that was not the primary reason for their referral" - AHP2 |
|  |  |  | "We get a really broad range of referrals and clients, so we don't get a high number of clients who have a diagnosis of cancer where that's their primary issue." - AHP3 |
|  |  |  | "Yes, most clients have a cancer history, it's quite common as a comorbidity" - HP4 |
|  | 1. Affordable and convenient access |  | "someone who doesn't have the right financial resources to be able to access these services easily, so if there is a CHS that does offer a range of services that can meet those needs and really pick them up and identify what those needs are, and connect them in to a service that's local and cost-effective to them." - AHP4 |
|  |  |  | "The vaste majority of our clients come from a low income, so if a client has financial hardship their fees can be waved." - AHP2 |
|  |  |  | "For people to access local services to where they live, for people to be aware of other services available within their local community health service." - AHP1 |
|  | 1. Consumer engagement-dependent | to improve overall health, access help, and regardless of disease type and stage | "Those interested in increasing fitness and becoming healthy in general and interested in increasing overall health post-treatment" - AHP1 |
|  |  |  | "Those that are able to identify and articulate their particular issues and are motivated to do something about it, but don’t know how to go on about accessing help or what sort of support that they need." - AHP2 |
|  |  |  | "clients that are able to clearly identify their own goals and are at that stage where they're ready to accept help and they're ready to work on those and make changes." - AHP3 |
|  |  |  | "People need to be open to this support, because an initial barrier would be a willingness to engage" - AHP4 |
|  |  |  | "the personal characteristics of the client, I guess, rather than the complexity of their medical history or diagnoses or treatment" - AHP3 |
|  |  |  | "So it was not the type of cancer they've had, but more about the recovery process." - AHP1 |
|  |  | Prevented by poor mental health, COVID-19 restrictions, and unstable disease | "people with neurological or cognitive issues as a result of their cancer dx/treatment that impact on their communication personality reasoning impulsivity. There's kind of a unique set of challenges that comes with those sorts of clients." - AHP2 |
|  |  |  | "I did mention there was a client that has been difficult to engage. They've also had some mental health comorbidities as well, so that certainly has impacted." - AHP3 |
|  |  |  | "There seems to be a hesitancy in over 65 years demographic due to fear of COVID-19" - AHP4 |
|  |  |  | "People can end up back in hospital, and then they’re back out in the community, so it can often be a little bit of stopping and starting with their treatment, with their therapies or Allied Health therapy in the community. So, I think that’s definitely a challenge if their condition is a bit more unstable" - AHP5 |
| 1. Sustainability of GL-CS | 1. Multi-level communication | To maintain relationship between acute and community care settings, and internally top-down and between teams | "getting the word out more about this program to perhaps the oncologists or the oncology nurses at the hospitals, and getting them to refer a little bit more, might actually help us to support that program more" - AHP5 |
|  |  |  | "we haven't had as much communication from our managers around the program ... so that's fallen on the care coordinators to do to educate the rest of the team about the program." - AHP3 |
|  |  |  | "I had assumed other discipline managers would also [communicate to their teams about the GL-CS program]. But I think that I was incorrect in assuming that... when we've done an internal referral, we've needed to provide the clinician with a bit of information about what this program is." - AHP3 |
|  | 1. Ongoing training | To increase ICC capacity and upskill ICC role | "it's good if there can be multiple staff involved in seeing this kind of clients, because it's nice to have someone else to be able to talk to and get their perspectives." - AHP2 |
|  |  |  | "if it were to continue, if it were to fit in with our organisation, we might need to have a care coordinator role based at each site so that you're able to be more efficient and have more knowledge of the different services in your area rather than just having someone based at one site." - AHP3 |
|  |  |  | "I would need ongoing professional development. " - AHP1 |
|  |  |  | "If more staff were going to be involved, training in cancer survivorship care would be beneficial." - AHP2 |
|  |  |  | "I think also further training on looking at - especially for the care coordinator role, looking at realistic goal setting and that motivational interviewing skills, I think that would also be really beneficial." - AHP3 |
|  | 1. Funding considerations | To streamline various CHS funding schemes | "In community health, we have a few different funding streams. Sometimes depending on the age of the client, it was a bit tricky to work out what the best funding stream to go with was, ... I think that was just something that was maybe a little bit confusing for the client to navigate and for us to work out." - AHP3 |
|  |  |  | "Each CHS has a slightly different funding source. So for us it has been about [figuring] out how to work things out for this program " - AHP4 |
|  |  |  | participants were supportive of maintaining the integrated care coordinator role at their local community health services, "but who’s going to pay for it – care coordination role? A big limitation would be funding this role." - AHP1 |
|  | 1. Workload management | To address staffing issues, workload allocation and administrative assistant needs | "need to address staffing issue " - AHP2 |
|  |  |  | "When we first started this program, we had 3 staff members, me another integrated care coordinator occupational therapist and an admin staff doing the initial paperwork. At the end of last year both members left, and they were not replaced. So that significantly increased my workload because I also have my own clients to see." - AHP2 |
|  |  |  | "getting them [clinicians] to refer a little bit more, might actually help us to support that program more so that we can actually allocate some time to it, within our week." - AHP5 |
|  |  |  | "Overtime we need to come up with a process that is efficient for us all." - AHP4 |
|  |  |  | "the only real issue that we faced was just, yeah, trying to fit those clients into our regular diaries with our regular clients as well." - AHP5 |
|  |  |  | "If the number of clients increased, then I would need more allocated hours to the program." - AHP1 |
|  |  |  | "you don't know how many referrals you're going to get or when they're going to come through so it's quite hard to plan to leave appointments open for them because you don't know when they're going to come through" - AHP2 |
|  |  |  | " I would probably have to block out extra time in my diary if we were to continue this moving forward, which can therefore put pressure on me seeing my other sort of dietician clients" - AHP5 |
|  |  |  | "they would definitely have to have the resources like an admin assistant" - AHP5 |
|  |  |  | "If the admin side of things could be completed by an assistant or outsource admin work to share the workload a bit" - AHP2 |
|  |  | Management support | "I've been very lucky that my manager at my workplace has been really supportive of taking on the Cancer Survivorship care coordinator role and so I've been able to fit that in with my existing KPIs for how many new clients I see per month and what my daily targets are." - AHP3 |
|  |  |  | "management need to work out funding model and efficient ways to process referrals, and how these clients in a funding streams are going to fit in this model" - AHP4 |
|  |  |  | "we would definitely have to get approval from our managers, and I think there would be a bit of push back there. They often like us to fill up our diaries as much as we can, and if that appointment time doesn’t get taken up, I guess that’s a missed appointment that someone else could have taken." - AHP5 |
|  | 1. Expansion |  | "there would be lots of clients in the Northern or Western regions that would definitely need that sort of support as well. So, it would be good to have community health centres around the whole of Victoria supporting this program" - AHP5 |
|  |  |  | "i would expand assessment tool to be a bit more holistic to include other risk factors; like alcohol use, smoking, where do you live/who do you live with/domestic violence, to cover a bit more like mental health." - AHP1 |
