# Supplementary material for: Implementation of a referral pathway for cancer survivors to access allied health services in the community
Source: BMC Health Serv Res. 2023 May 4;23:440. doi: 10.1186/s12913-023-09425-4 (PMC10159668; doi:10.1186/s12913-023-09425-4)
Supplement: Supplementary file 4 — Supplementary Material 4 [file 12913_2023_9425_MOESM4_ESM.docx]

**Supplemental material 4 - Thematic summary of hospital-based Health Professionals interviews**

| **Theme** | **Sub-theme** | **Code** | **Quote** |
| --- | --- | --- | --- |
| 1. A holistic and complementary model of care | Continuity of cancer survivorship care | Holistic | "the whole idea of community health is to actually have that holistic care so that they’ve got options to – a range of services that they may not think that they actually need" EH1 |
|  |  |  | "[this is] another step...in their recovery" EH2 |
|  |  |  | “I’ve also got a couple in because they’ve wanted to have some podiatry done to their feet and I thought it was a way of getting them involved and hopefully be able to explore other aspects” EH5 |
|  |  |  | "We have to look at avenues where we can provide care for them long – way past their treatment and way past anything else, because most of our patients are kind of cured, we would hope, but unfortunately, some of them do still suffer from consequences of their treatment, so I think it’s really essential." EH1 |
|  |  | Flexible and convenient | "People who don’t want to attend in a group and people who don’t want the whole realm of services, just want one or two, it’s much more appropriate for them. Also, the fact that it’s at various locations is fantastic." EH2 |
|  |  |  | "Proximity [to a health service may be an issue]. But I guess that barrier has been largely taken down by getting other community health organisations onboard." EH5 |
|  |  | Supportive | "It's no good them coming to us week by week and they're struggling, they're not coping, they're not coping with the symptoms of their treatment. A lot of them complain of fatigue. So to be able to have that support for them to get over that, you know, cross the line to get them to that point is really, really good." EH5 |
|  |  | Complementary | "I’ve had a positive experience, and I think it’s really good to have something there that we can refer onto when we don’t have those services ourselves. " EH6 |
|  | Additional services need | Smoking cessation support | "I’ve had a couple of patients who really need support in stopping smoking and Carrington don’t have a specific smoking sensation program. QUIT doesn’t help – doesn’t suit everybody. " EH3 |
|  |  | Social worker | "a lot of our patients, they might have to stop working, and they might not – they’re going through a tough time. They might not have supports and things available. I think for a cancer patient, having the – having a social worker available is probably one of the most important services that we could give them, and we just don’t have that." EH6 |
|  |  |  | "The only thing that I wish that they offered that they don’t is social work" EH 7 |
|  |  | Accessing issues | "there was limited access to a counselling service and I think there was staff changes or no staff. I had one patient that was really struggling to try and get some counselling through community health " EH1 |
| 2. The referral process | Easy |  | "It’s a very easy to use tool, I think. The one page is essential for health professionals to just make it a really easy tick sheet and then you can just add other information. " EH1 |
|  |  |  | "It was actually easy and I did most of it online, so I just ticked and did whatever I could online, just fill in the boxes and then printed it, signed it and then rescanned it in. So I actually found that really easy. " EH5 |
|  | Sharing patient information | Cautious | "I sometimes struggle to know what to include and confidentiality wise, I guess, or confident privacy I guess, yeah. I don’t want to include stuff unless it’s necessary. " EH2 |
|  |  |  | "I only provide that information if the physio or [project manager] has actually asked for it. " EH1 |
|  |  | Guided by community health services | "In the past, if I haven’t filled out anything correctly or there’s been any issues, I’ve had communication back quite quickly – needing all the right information " EH6 |
|  |  |  | "I would just refer them on, and then it would be up to the team to get in touch with me should they be refusing a patient or think that a patient’s not the right fit for their program. " EH7 |
|  |  | Communication flow between settings | "If I’ve got a problem, I normally call as well. I call [the project manager] and we talk through an issue or a complication or a query. " EH7 |
|  |  |  | "I’ve always had good communication from [community health services] after I’ve sent a referral " EH6 |
|  | Streamlining considerations | using existing system | "you can probably streamline it if you're able to add it to the referral system into CPF " EH5 |
|  |  | central point of contact | "there would have to be a common email address wouldn't there, whether it be the Cancer Survivorship email address that you could actually just email all referrals to I would think, streamlining it so that you've got one point of contact for all referrals" EH5 |
|  |  |  | "All the referrals go to the one person, the intake person, who then streamlines them and sends them out to the appropriate people. Whether you had something like that might work." EH5 |
|  | Broadening eligible criteria |  | "I would like to refer people having active chemotherapy. I’ve got a lady, a 40-year-old now who’s incredibly affected by her breast cancer chemo and it would be great for her to have a physio who she could ring to have a simple rehab referral" EH2 |
|  |  |  | "sometimes we do immunotherapy type infusions, and they come every month or couple of weeks, and that might just be ongoing. So, if we had the availability to refer these patients on as well, I think that would be helpful" EH6 |
|  |  |  | "We do have a lot of patients who are quite stable, but who will be on long-term treatments. So, I guess those sorts of patients would be great to be able to refer as well" EH6 |
|  | Impact of COVID-19 | Service disruptions | "I think COVID has been very disruptive, created uncertainty rather than service being reduced" EH4 |
|  |  |  | "with COVID too, everything is just so – there's so much more to do with patients, it's added a lot of stresses too." EH5 |
|  |  | Re-prioritised services | "this COVID time in acute health, everything seems to be about cutting back and not providing excess service" EH2 |
|  |  |  | "you’re just trying to keep your head above water during COVID and making sure your patients are getting cared for, so sometimes those referrals to community health just kind of go out the window" EH1 |
|  |  |  | "some services have been reduced. As I said, the waiting list for example for OTs and to do home assessments and – yeah, I know psychology – yeah, there’s a lot of services that I have experienced have been reduced" EH2 |
|  |  |  | "you've got staff that are really busy on the wards… and the last thing they think of is am I going to refer this person?" EH5 |
|  |  | Telehealth | "you’re doing a lot with telehealth where you don’t get those cues that you normally get when you’re in the clinic, that you think that there’s something not right with patients" EH2 |
|  |  |  | "We don’t pick up a lot of the cues when things are done over the phone. Face-to-face, you do pick up a lot more" EH3 |
| 3. A patient-driven process | A central role |  | "it's really whether they accept it or not… so as long as they're receptive to [the referral], as long as they're willing to partake in [the program]" EH5 |
|  |  |  | "you could argue that the cohort, whether if they’ve had previous community - previous interaction with community or council services that haven’t gone in a positive manner. That would also lead them to be wanting to - or potentially declining [other community services]" EH7 |
|  | Sex difference |  | "it’s interesting that sometimes the women are more likely to engage in wanting to be referred, but I find men are not wanting to be referred as often" EH3 |
|  |  |  | "I actually find that men are not very receptive – for really anything. So I think it's just a matter of getting men more onboard. I don't know how to change that. Maybe it's something that we do, but I actually find and even in my previous role, men are not very good at talking. Men are not very good at accepting help" EH5 |
|  | Role of carers |  | "You tend to have to get into the ear of their [patient's] partners to say how beneficial it is" EH1 |
|  | Stage of recovery |  | "Some people just don’t find that it’s the right fit at the right time for them" EH7 |
|  |  |  | "I think they don’t normally like to be referred early on" EH1 |
|  |  |  | "Their cancer maybe just starting to progress or they’re getting symptoms" EH7 |
|  |  |  | "They [patients] may accept a referral when they’re at their wits end" EH1 |
|  |  |  | "Until they’re at crisis point, they [patients with complex disease burden] often won’t accept the service or the help" EH7 |
|  | With a purpose |  | "it's trying to encourage them that this is actually a really good service for them" EH5 |
|  |  |  | "just to say that improved outcomes from being referred to specific programs, just to back it up a little bit" EH3 |
|  |  |  | "we approached him and we said look, this can help you as far as maybe exercise, maybe with your food, a little bit more ongoing support with this cancer diagnosis out in the community. Because whilst we can provide that support for him for his treatment, once he goes home we're not there" EH5 |
| 4. Promoting GL-CS | Raise awareness | in acute settings | "I didn’t know about the new areas that it was operating in and it would have been good to have been able to inform the person I referred about those before she was contacted sort of thing. Yeah. I don’t know, a way of keeping us in the loop." EH2 |
|  |  |  | "Making us aware of it is a good idea." EH3 |
|  |  |  | "[Before joining this project] I didn't know what services are around and wasn't sure what the hospital had in place for [cancer survivors]. So I guess from that point of view they probably missed out" EH5 |
|  |  | in the community | "promote the program through either support groups or GPs… I think we need to get the message out to the wider community, not just at hospital setting." EH1 |
|  |  | in patients | "inform [patients] about survivorship courses, rehab… " EH2 |
|  |  |  | "[inform patients about] what’s available to them in the community, because a lot of patients probably don’t know that " EH1 |
|  |  | by patients | "I had a patient last week telling me of what a wonderful program it was and she told me all the services and I must say, it woke me up again. " EH2 |
|  |  |  | "I did have one gentleman who gave some really good feedback and was really grateful of the referral and had a very successful outcome with them. He ended up doing some physio, and that worked very well. " EH6 |
|  | Maintain awareness in acute settings | Communication strategies | "I think even an ongoing newsletter or an update every couple of months or every three months about how it’s going, just to remind us if we’ve forgotten " EH2 |
|  |  |  | "a newsletter to people who refer might be a good idea. Maybe even keeping a number of, we’ve received so many referrals this month. Just so it’s a – it raises people’s awareness of, gee, I should have referred that person. " EH3 |
|  |  |  | "I think there needs to be ongoing education, ongoing reminders that this service is actually available for patients " EH5 |
|  |  |  | "I think sometimes people probably maybe forget about these different services and things like that. So, just a – maybe a regular e-mail or something from [community health services] just to remind people that they are there, and they are willing to take on these patients. " EH6 |
|  |  |  | "I think putting the program out there regularly and always being in people’s faces or the doctors’ faces reminding them of the service. I think that is the best way to ensure that the program’s sustainable. " EH7 |
|  |  | Be pragmatic | "To be honest, if it was via email, I don’t know - I mean I don’t think even half the people would read it. " EH7 |
|  |  | Patient feedback | "[I ask my patients] can you just keep us informed how you're finding it, whether you're…enjoying it, the benefits? Because that's sort of information that I also need to be able to say to our other patients, look, we've had this person, he actually found it really beneficial for these reasons. This might actually work for you as well. " EH5 |
|  |  |  | "I’ve referred a few patients to the program. I’ve only had positive feedback " EH1 |
|  | Increase knowledge | of cancer survivorship | "I think concept of survivorship will take quite some time to get credibility, so we need to persevere. " EH4 |
|  |  | of services available by GL-CS | "the last few referrals I’ve made have probably been in error of myself thinking that there was some social work and some other supports available, which wasn’t the case. " EH6 |
|  |  |  | "there are things that I ask of [the project manager] that I, literally, have no idea about. Through her experience within the Carrington or Health Ability organisation, she not only knows, not just what the Good Heath or Cancer Survivorship program can provide, but also things outside the square as well. " EH7 |
